# Inferring Homologous Recombination Deficiency of Ovarian Cancer From the Landscape of Copy Number Variation at Subchromosomal and Genetic Resolutions

**DOI:** 10.3389/fonc.2021.772604

**Published:** 2021-12-16

**Authors:** Meng Zhang, Si-Cong Ma, Jia-Le Tan, Jian Wang, Xue Bai, Zhong-Yi Dong, Qing-Xue Zhang

**Affiliations:** ^1^ Department of Obstetrics and Gynecology, Reproductive Medicine Centre, Sun Yat-Sen Memorial Hospital, Sun Yat-Sen University, Guangzhou, China; ^2^ Guangdong Provincial Key Laboratory of Malignant Tumor Epigenetics and Gene Regulation, Medical Research Center, Sun Yat-Sen Memorial Hospital, Sun Yat-Sen University, Guangzhou, China; ^3^ Department of Radiation Oncology, Nanfang Hospital, Southern Medical University, Guangzhou, China; ^4^ Information Management and Big Data Center, Nanfang Hospital, Southern Medical University, Guangzhou, China

**Keywords:** homologous recombination deficiency, copy number variation, ovarian cancer, biomarker, chromosome

## Abstract

**Background:**

Homologous recombination deficiency (HRD) is characterized by overall genomic instability and has emerged as an indispensable therapeutic target across various tumor types, particularly in ovarian cancer (OV). Unfortunately, current detection assays are far from perfect for identifying every HRD patient. The purpose of this study was to infer HRD from the landscape of copy number variation (CNV).

**Methods:**

Genome-wide CNV landscape was measured in OV patients from the Australian Ovarian Cancer Study (AOCS) clinical cohort and >10,000 patients across 33 tumor types from The Cancer Genome Atlas (TCGA). HRD-predictive CNVs at subchromosomal resolution were identified through exploratory analysis depicting the CNV landscape of HRD *versus* non-HRD OV patients and independently validated using TCGA and AOCS cohorts. Gene-level CNVs were further analyzed to explore their potential predictive significance for HRD across tumor types at genetic resolution.

**Results:**

At subchromosomal resolution, 8q24.2 amplification and 5q13.2 deletion were predominantly witnessed in HRD patients (both *p* < 0.0001), whereas 19q12 amplification occurred mainly in non-HRD patients (*p* < 0.0001), compared with their corresponding counterparts within TCGA-OV. The predictive significance of 8q24.2 amplification (*p* < 0.0001), 5q13.2 deletion (*p* = 0.0056), and 19q12 amplification (*p* = 0.0034) was externally validated within AOCS. Remarkably, pan-cancer analysis confirmed a cross-tumor predictive role of 8q24.2 amplification for HRD (*p* < 0.0001). Further analysis of CNV in 8q24.2 at genetic resolution revealed that amplifications of the oncogenes, *MYC* (*p* = 0.0001) and *NDRG1* (*p* = 0.0004), located on this fragment were also associated with HRD in a pan-cancer manner.

**Conclusions:**

The CNV landscape serves as a generalized predictor of HRD in cancer patients not limited to OV. The detection of CNV at subchromosomal or genetic resolution could aid in the personalized treatment of HRD patients.

## Introduction

Homologous recombination deficiency (HRD), a driving factor of tumorigenesis, can lead to damage in the repair process of DNA double-strand breaks (1) and the resultant genomic instability (2), a common feature of many tumors but is mainly seen in ovarian cancer (OV) (1). Recently, researchers have shown an increased interest in HRD because of its important role in chemotherapy, targeted therapy, and immunotherapy (3, 4). Cytotoxic agents such as platinum analogues (3, 5), topoisomerase I inhibitors, topoisomerase II inhibitors (6, 7), and anti-metabolite gemcitabine (8) are effective in HR-deficient tumors. HR-deficient cells are also extremely sensitive to poly-ADP ribose polymerase (PARP) inhibitors, which target DNA damage response and show synthetic lethality when applied to HR-deficient cells (9, 10). In addition, tumors with HRD are considered more immunogenic than tumors without genetic defects in the HR pathway, making them potential candidates for immune checkpoint blockade (4). Therefore, accurate monitoring of HRD is clinically important as it indicates sensitivity to DNA damage agents and PARP inhibitor therapy, which helps to personalize and limit the drug use to the beneficiary population, avoiding unnecessary toxicity to those who will not benefit.

Considerable efforts have been made to identify and develop effective predictive biomarkers; however, HRD identification in tumors remains controversial (11). In terms of the causes of HRD, biomarkers include mutations related to homologous recombination repair (HRR) pathway genes, such as *BRCA1/2* germline and somatic mutations (12, 13), *BRCA1* promoter methylation (3), and mutations of other genes in HRR (e.g., *RAD51C*, *CHEK2*, *BRIP1* (14), and *PTEN*) (15, 16). In terms of the outcome of HRD, three single-nucleotide polymorphism-based biomarkers to quantify the extent of chromosomal abnormality, telomeric-allelic imbalance (TAI) (17), loss of heterozygosity (LOH) (18), and large-scale state transitions (LST) (19), were significantly associated with *BRCA1/2* status, and the combined TAI, LOH, and LST scores were retrospectively validated to predict the response to platinum-containing neoadjuvant chemotherapy (20). However, somatic reverse mutations that render functionally proficient homologous recombination and result in false positives (21–23) still limit the clinical application of existing markers, highlighting the urgent need to discover more effective and accurate biomarkers.

Gross chromosomal rearrangements and overall genomic instability are increased in HR-deficient cells (2, 24), which are the main cause of copy number variations (CNVs) (25). HR-deficient cells either have difficulty repairing DNA damage and thus progress to some form of programmed cell death, or attempt to repair DNA damage using processes such as nonhomologous end-joining and thus generate CNVs (25–27). Array comparative genomic hybridization (aCGH) is a technique for detecting CNVs in tumor genomes (28), and aCGH-based genomic scar analysis showed that the presence of *BRCA*-like aCGH signals predicted a preferential response to platinum-based drugs (29). Altogether, previous studies suggest that there may be a correlation between CNVs and HRD status of patients; however, little is known about CNVs of which specific genomic loci in tumors are directly associated with HRD.

In this study, through the analysis of the CNV landscape in patients from the Australian Ovarian Cancer Study (AOCS) cohort and The Cancer Genome Atlas (TCGA), we found that the CNVs of three specific chromosomal fragments in OV were significantly correlated with HRD, and the correlation between the CNV of 8q24.2 and HRD was verified across tumor types. In addition, *MYC* and *NDRG1* genes located at this locus were also associated with HRD in a pan-cancer manner. Our study enriches HRD biomarkers at subchromosomal and genetic resolutions, and provides a new perspective for the cross-tumor study of HRD status in patients.

## Materials and Methods

### Patient Cohorts

#### TCGA-OV Cohort

CNV data of 587 patients with OV were retrieved from TCGA at https://www.cancer.gov/tcga (**Table S1**). The majority of patients in the TCGA-OV cohort were Caucasian (84.77%), diagnosed with serous cystadenocarcinoma (98.92%), and within stage III (76.34%) (**Table S2**). HRD score of each TCGA sample, retrieved from the UCSC Xena platform at http://xena.ucsc.edu/ (30), was the unweighted sum of TAI, LOH, and LST counts (**Table S3**) (20). TAI was defined as the number of regions with an allelic imbalance that extend into the subtelomere but not across the centromere (17). LOH was defined as the count of chromosomal regions with loss of heterozygosity, shorter than the entire chromosome and longer than 15 Mb (18). LST was defined as chromosomal breakpoints between adjacent regions of at least 10 Mb each after smoothing and filtering small-scale CNVs shorter than 3 Mb (19). Accordingly, 558 OV patients with matched CNV and HRD score data were divided into the HRD group (HRD score ≥ 42) and the non-HRD group (HRD score < 42) using the cut-point determined in a previous study (**Table S3**) (20).

#### AOCS Cohort

The AOCS cohort included 93 patients with stage III (84.95%) or stage IV (15.05%) epithelial ovarian, primary peritoneal, or fallopian tube cancer, who received platinum-based chemotherapy as part of the primary treatment (**Table S1**). CNV data were available from the International Cancer Genome Consortium (ICGC) at https://dcc.icgc.org/. HRD status was available for 80 patients in the AOCS cohort according to HRR gene mutations; patients with any of the *BRCA1/2* germline or somatic mutations, *BRCA1*, *RAD51C* promoter methylation, *CHEK2* mutation, *BRIP1* mutation, *RAD51C* mutation, and somatic *PTEN* deletion were assigned to the HRD group, and patients without all of the above molecular characteristics were assigned to the non-HRD group (**Table S4**) (31).

#### TCGA Pan-Cancer Cohort

CNV data of 11,167 TCGA patients from 33 cancer types were obtained from the Genomic Data Commons (GDC) data portal (**Table S1**). The clinical characteristics of the TCGA pan-cancer cohort are summarized in **Table S5**. As in the TCGA-OV cohort, 10,560 patients with matched CNV, clinical, and HRD score data were divided into HRD (HRD score ≥ 42) and non-HRD (HRD score < 42) groups (**Tables S3**, **S6**).

### Study Design

This study proposed and validated new biomarkers of HRD from subchromosomal to genetic resolutions (**Figure S1**). Exploratory analysis depicting the CNV landscape of HRD *versus* non-HRD patients in the TCGA-OV cohort was carried out to identify several CNVs that were predictive of HRD in patients with OV, which was further validated in the TCGA-OV and AOCS cohorts. Later, we explored whether the above CNVs and genes at the corresponding locus could predict HRD across cancer types by studying the TCGA pan-cancer cohort.

### Depiction of Genome-Wide CNV Landscape

Genome-wide CNV landscape at subchromosomal and genetic resolutions was measured using GISTIC 2.0 (genomic identification of significant targets in cancer 2.0) (32), which has been widely used for CNV quantification of chromosomal fragments and genes in multiple cancer types (33, 34). Copy number segment files, excluding regions within 3 Mb around the centromeres and within 1 Mb at the ends of chromosomes, were input into GISTIC 2.0. Genomic regions significantly amplified or deleted in a set of samples were identified, with a G-score assigned for each aberration considering both the amplitude and frequency. In addition, a gene GISTIC algorithm was used to measure CNVs at the genetic level. GISTIC 2.0 considers chromosomal fragments or genes to be undergoing homozygous deletion, single copy deletion, low-level amplification, or high-level amplification if the corresponding GISTIC call = −2, −1, 1, or 2, respectively.

### Statistical Analysis

Statistical analyses were performed using GraphPad Prism (version 8.0.1) and R (version 3.6.1). The receiver operating characteristic (ROC) curve was applied to test the correlation between HRD and copy number values; the area under the ROC curve (AUC) is a commonly used summary measure of discriminatory accuracy, ranging from 0.5 (random guess) to 1 (perfect discrimination). Non-parametric statistical tests were used in the differential and correlation analyses throughout the study because the data did not meet the assumption for normality (**Figure S2**). Copy number values of the HRD and non-HRD groups were compared using the Wilcoxon signed rank test. LOH, TAI, and LST among CNV groups were compared using the Kruskal–Wallis test, and pairwise comparisons were conducted using the Wilcoxon signed rank test. The Spearman’s rank correlation was used to validate the pan-cancer correlation between gene-level CNVs and HRD. The false discovery rate (FDR) *q*-value was estimated to represent the false-positive probability. *p* ≤ 0.05 in an individual hypothesis test or *q* ≤ 0.05 in large-scale multiple testing was considered statistically significant.

## Results

### Predictive Role of CNVs for HRD in OV

**Figure 1:**
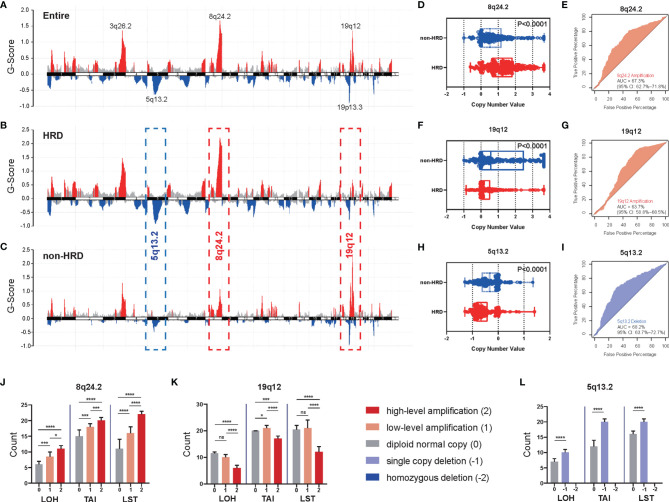
The CNVs of specific chromosomal fragments associated with HRD status in ovarian cancer. **(A)** The enrichment score of genome copy number variations (CNVs) in all ovarian cancer patients from the TCGA dataset. **(B**–**I)** According to HRD score, patients with ovarian cancer were divided into an HRD group (HRD score ≥ 42) and a non-HRD group (HRD score < 42). **(B)** Enrichment score of CNVs in the HRD group. **(C)** Enrichment score of CNVs in the non-HRD group. Red: amplification, blue: deletion. **(D, F, H)** Differences of copy number value of 8q24.2 **(D)**, 19.12 **(F)**, and 5q13.2 **(H)** calculated by GISTIC between the HRD group and the non-HRD group. **(E, G, I)** Performance of 8q24.2 amplification **(E)**, 19q12 amplification **(G)**, and 5q13.2 deletion **(I)** in predicting HRD/non-HRD. **(J–L)** According to the cutoff given by GISTIC 2.0, we divided the copy number variation states of 8q24.2 **(J)**, 19q12 **(K)**, and 5q13.2 **(L)** into five categories: high-level amplification (red), low-level amplification (orange), diploid normal copy (gray), single copy deletion (purple) and homozygous deletion (blue), and explored the relationship between them and the three constituent indexes of HRD score—LOH, TAI, and LST. Statistical analysis was performed by Wilcoxon signed rank test. Significant levels: ns, not significant; *p < 0.05; ***p < 0.001, ****p < 0.0001.

Given the high frequency of HRD in OV, we first analyzed the CNVs of chromosomal fragments in the TCGA-OV cohort and found significant amplifications of chromosomes 3q26.2, 8q24.2, and 19q12 in OV patients, while chromosomes 5q13.2 and 19p13.3 were significantly deleted (**Figure 1A**). To further investigate the relationship between CNVs of these chromosomal fragments and the HRD status of patients, we analyzed CNVs in HRD patients *versus* non-HRD patients, and found that 5q13.2 was significantly deleted and 8q24.2 was markedly amplified in the HRD group compared with the non-HRD group, while 19q12 was remarkably amplified in the non-HRD group (**Figures 1B, C**). Based on these findings, we hypothesized that CNVs of the three fragments 5q13.2, 8q24.2, and 19q12 could predict HRD status in OV patients. Therefore, we compared the copy number values between patients in the HRD and non-HRD groups and explored whether the CNVs of 5q13.2, 8q24.2, and 19q12 could serve as potential predictors of HRD status. The results revealed that patients with HRD were prone to obtain a higher amplification value of 8q24.2 (**Figure 1D**, *p* < 0.0001, Wilcoxon signed rank test) and deletion value of 5q13.2 (**Figure 1H**, *p* < 0.0001, Wilcoxon signed rank test) than those in the non-HRD group, with AUCs of 67.3% (**Figure 1E**) and 68.2% (**Figure 1I**), respectively. Notably, patients in the non-HRD group demonstrated a higher amplification value of 19q12 than those in the HRD group (**Figure 1F**, *p* < 0.0001, Wilcoxon signed rank test) with an AUC of 63.7% (**Figure 1G**), suggesting that CNVs of these three chromosomal fragments may be effective predictors of HRD status.

To further confirm the predictive value of CNVs of the three fragments for HRD, we explored the relationship between the deletion or amplification of 5q13.2, 8q24.2, and 19q12 and three components of HRD score, LOH, TAI, and LST (20). The results indicated that significant differences in LOH, TAI, and LST existed among groups stratified by the degree of 8q24.2 amplification (**Figure 1J**, all *p*<0.0001, Kruskal–Wallis test); pairwise comparisons (**Table S7**) indicated that the greater the degree of 8q24.2 amplification, the higher the number of LOH, TAI, and LST (high-level amplification vs. low-level amplification: all *p* < 0.05, Wilcoxon signed rank test; low-level amplification vs. diploid normal copy: all *p* < 0.05, Wilcoxon signed rank test). In addition, significant differences in LOH, TAI, and LST were observed among groups concerning 19q12 amplification (**Figure 1K**, all *p*<0.0001, Kruskal–Wallis test); however, LOH, TAI, and LST were mainly upregulated in high-level amplification (**Table S7**, all *p* < 0.05, Wilcoxon signed rank test), whereas LOH and LST were generally similar between diploid normal copy and low-level amplification (**Table S7**, *p* > 0.05, Wilcoxon signed rank test). Regarding 5q13.2, single copy deletion of this fragment yielded significantly higher LOH, TAI, and LST than the diploid normal copy (**Figure 1L**, all *p*<0.0001, Wilcoxon signed rank test), but no homozygous deletion was detected. Taken together, these results suggest that the CNVs of these three fragments can be used to predict HRD status in OV.

### Validation of the Predictive Value of CNVs for HRD in AOCS Cohort

**Figure 2:**
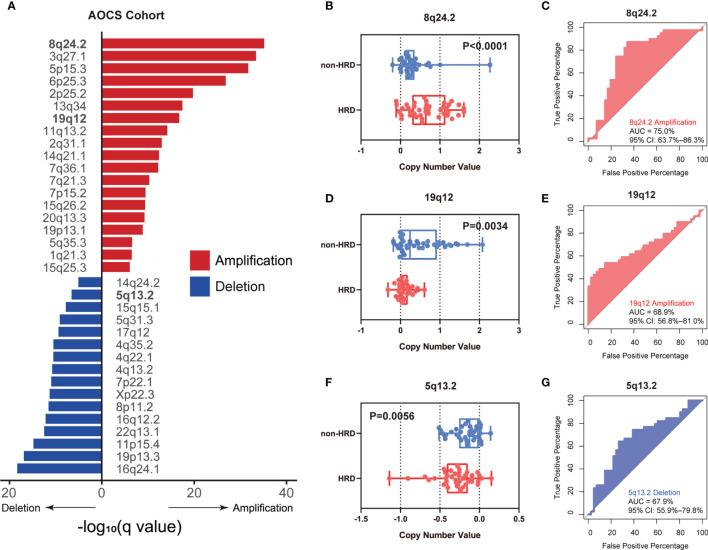
External validation of the CNV biomarkers for HRD status in AOCS. **(A)** The enrichment level of genome CNVs in all ovarian cancer patients from the AOCS cohort. **(B–G)** According to *BRCA1/2* mutation, *BRCA1* promoter methylation, and other HRR gene mutations (*RAD51C*, *CHEK2*, *BRIP1*, and *PTEN*), ovarian cancer patients in the AOCS cohort were divided into an HRD group and a non-HRD group. **(B, D, F)** Differences of copy number value of 8q24.2 **(B)**, 19q12 **(D)**, and 5q13.2 **(F)** calculated by GISTIC between HRD group and non-HRD group. **(C, E, G)** Performance of 8q24.2 amplification **(C)**, 19q12 amplification **(E)**, and 5q13.2 deletion **(G)** in predicting HRD/non-HRD. Statistical analysis was performed by Wilcoxon signed rank test in **(B, D, F)**.

To further verify our hypothesis about the predictive value of CNVs, external validation in another ovarian cancer cohort, AOCS (31), was carried out. CNVs of the three chromosomal fragments 8q24.2, 19q12, and 5q13.2 identified in TCGA-OV also showed significant enrichment in this cohort (**Figure 2A**, FDR *q*-value < 0.05). As expected, we observed significantly higher amplification of 8q24.2 (**Figure 2B**, *p* < 0.0001, Wilcoxon signed rank test) and deletion value of 5q13.2 (**Figure 2F**, *p* = 0.0056, Wilcoxon signed rank test) in the HRD group than in the non-HRD group, with AUCs of 75.0% (**Figure 2C**) and 67.9% (**Figure 2G**), respectively. The amplification value of 19q12 in the non-HRD group was significantly higher than that in the HRD group (**Figure 2D**, *p* = 0.0034), with an AUC value of 68.9% (**Figure 2E**). These results further provide robust evidence that the CNVs of 5q13.2, 8q24.2, and 19q12 may serve as effective biomarkers for HRD in OV.

### Applicability of CNVs of 8q24.2 in Predicting HRD Across Cancer Types

**Figure 3:**
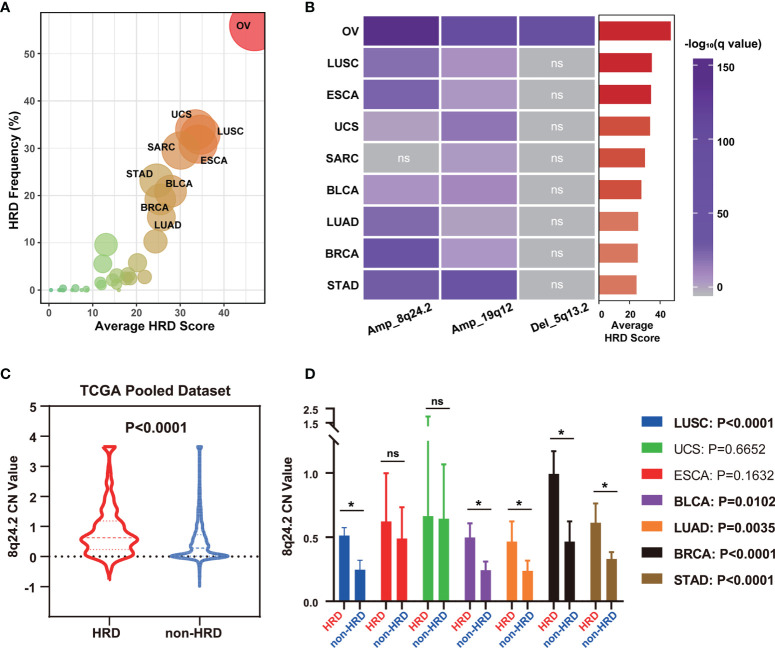
8q24.2 amplification for the prediction of HRD status in patients across cancer types. **(A)** The average HRD score and HRD frequency (≥42 as cutoff value) of 33 kinds of tumors in the TCGA dataset. **(B)** The CNV enrichment level of amplification of 8q24.2, 19q12, and deletion of 5q13.2 in nine kinds of tumors with the highest average HRD score. **(C, D)** According to HRD score, all tumor patients in TCGA were divided into an HRD group (HRD score ≥ 42) and a non-HRD group (HRD score < 42). **(C)** Violin plot of copy number value of 8q24.2 in the HRD group and the non-HRD group in all TCGA tumors. **(D)** Copy number value of 8q24.2 in the HRD group and the non-HRD group in 7 kinds of tumors. OV, ovarian serous cystadenocarcinoma. UCS, uterine carcinosarcoma. LUSC, lung squamous cell carcinoma. SARC, sarcoma. ESCA, esophageal carcinoma. STAD, stomach adenocarcinoma. BLCA, bladder urothelial carcinoma. BRCA, breast invasive carcinoma. LUAD, lung adenocarcinoma. Statistical analysis was performed by Wilcoxon signed rank test in **(C, D)** NS, not significant, *p < 0.05.

As HRD is a common feature of many tumors, such as ovarian, breast, and prostate cancers (1, 2), we investigated whether CNVs of 5q13.2, 8q24.2, and 19q12 for HRD status prediction apply to tumors other than OV. We first calculated the mean HRD scores of 33 tumors in the TCGA dataset to obtain HRD status distribution in different tumors and found that patients with ovarian serous cystadenocarcinoma, uterine carcinosarcoma, lung squamous cell carcinoma, sarcoma, esophageal carcinoma, stomach adenocarcinoma, bladder urothelial carcinoma, breast invasive carcinoma, and lung adenocarcinoma showed predominantly high mean HRD scores, while the lowest was witnessed in patients with thyroid carcinoma and acute myeloid leukemia (**Figure 3A** and **Table S5**). To investigate whether CNVs can be used for pan-cancer HRD detection, we explored the CNV frequencies of 8q24.2 and 19q12 amplifications and 5q13.2 deletion in nine types of tumors with the highest HRD scores (**Figure 3B**). In these cancers, 8q24.2 amplification was generally enriched, except for sarcoma, while 19q12 amplification most frequently occurred in ovarian serous cystadenocarcinoma and stomach adenocarcinoma. However, 5q13.2 deletion was only reflected in ovarian serous cystadenocarcinoma, suggesting that 8q24.2 amplification may be pan-cancer in the prediction of HRD and 5q13.2 deletion may be specific to OV. We then focused on the 8q24.2 and found that there was a significantly higher copy number value of this fragment in patients with HRD compared with the non-HRD counterparts in the TCGA pan-cancer cohort (**Figure 3C**, *p* < 0.0001, Wilcoxon signed rank test), which was confirmed in lung squamous cell carcinoma (*p* < 0.0001), bladder urothelial carcinoma (*p* = 0.0102), lung adenocarcinoma (*p* = 0.0035), breast invasive carcinoma (*p* < 0.0001), and stomach adenocarcinoma (*p* < 0.0001), respectively (**Figure 3D**, Wilcoxon signed rank test). Altogether, these results provide insights into the predictive role of the CNV of 8q24.2 for HRD across cancer types.

### Predictive Utility of *MYC* and *NDRG1* Amplifications for HRD Across Cancer Types

**Figure 4:**
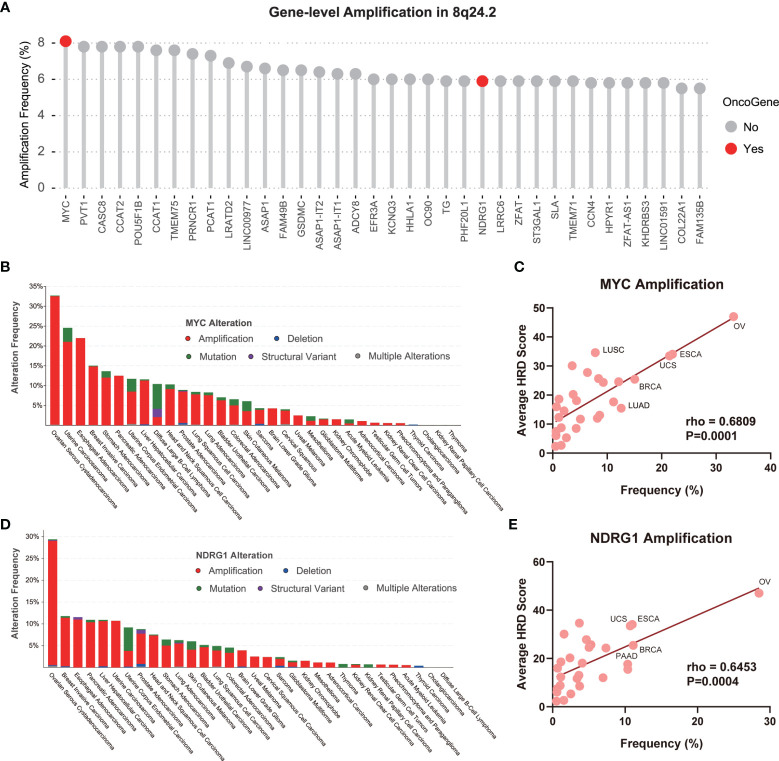
*MYC* and *NDRG1* amplifications as gene-level CNV biomarkers for pan-cancer HRD detection. **(A)** The amplification of the gene-level CNA of 8q24.2 in TCGA. Red: oncogene, gray: non-oncogene. **(B)** The alteration type and frequency of *MYC* in various tumors, red: amplification, blue: deletion, green: mutation, purple: structural variant, gray: multiple alterations. **(C)** The linear regression graph of the amplification frequency of *MYC* and the average HRD score of tumors. **(D)** The alteration type and frequency of *NDRG1* in various tumors. **(E)** The linear regression graph of the amplification frequency of *NDRG1* and the average HRD score of tumors. Statistical analysis was performed by Spearman test in **(C, E)**.

Similar to *BRCA1/2* playing a crucial role in the HRR process (2), the satisfactory performance of the CNV of 8q24.2 in predicting pan-cancer HRD led us to speculate that some genes on this fragment may influence the HRD status of patients through an intrinsic mechanism. Therefore, an analysis of gene amplification frequency in 8q24.2 using the TCGA pan-cancer cohort was performed to determine the correlation between genes and HRD. As illustrated in **Figure 4A**, genes within 8q24.2 were frequently amplified across tumors, including oncogenes *MYC* and *NDRG1*. Given the close association of oncogenes with therapy and tumor biology (35), we attempted to gain an insight into the potential value of *MYC* and *NDRG1* in recognizing HRD. Intriguingly, we counted the type and frequency of *MYC* and *NDRG1* alterations in all TCGA tumors and found that the main alteration type of *MYC* and *NDRG1* was exactly amplification. In addition, the tumors showing the highest alteration frequencies of *MYC* and *NDRG1* were both ovarian cancers, whereas the frequencies in thyroid carcinoma were lower, which is consistent with the highest HRD scores for ovarian cancers and low for thyroid cancers in **Figures 3A**, **4B, D**). This result implied that *MYC* and *NDRG1* amplifications might be directly related to HRD. We then investigated the relationship of *MYC* and *NDRG1* amplifications with the HRD score using pan-cancer data, and the results were per our hypothesis: *MYC* and *NDRG1* amplifications are significantly correlated with HRD score (*MYC*: rho = 0.6809, *p* = 0.0001; *NDRG1*: rho = 0.6453, *p* = 0.0004; **Figures 4C, E**; Spearman’s rank correlation). These results indicate that *MYC* or *NDRG1* amplification might potentially serve as a novel and pan-cancer biomarker of HRD.

## Discussion

HRD is a common feature among many tumors (1) and an important therapeutic target (3). However, HRD biomarkers are diverse and controversial (11). Currently, the only patient stratification criterion, *BRCA1/2* germline mutation, has its limitations. On the one hand, some tumors carrying *BRCA1/2* germline mutations are not sensitive to PARP inhibitors (36, 37); on the other hand, patients lacking *BRCA1/2* germline mutations sometimes benefit from PARP inhibitors in combination with DNA damage agents (38). Therefore, patients may receive incorrect treatment or miss treatment opportunities. What makes it even more difficult is that current assays based on single locus are largely insensitive to the reversion of HRD, which restores *BRCA1/2* and HR function and consequently results in false positives. In addition, previous studies have focused on genes that play a role in HRR, but each tumor type is different in the underlying mechanisms of HRD, which may eventually be defined by a set of biomarkers (39). Therefore, there is an urgent need to identify uniform biomarkers of HRD to detect potential defects in HR shared by all these different mechanisms. In this regard, efforts to establish effective biomarkers from a higher dimension (e.g., CNV at subchromosomal level) beyond point mutation are warranted to complement the field and aid in the personalized treatment of HRD patients.

Generally, CNV refers to deletion or amplification in the copy number of genomic segments (>1 kb) at the submicroscopic level. CNV formation mechanisms include non-allelic homologous recombination, non-homologous end-joining (25), and fork stalling and template switching mechanisms based on DNA misreplication (40, 41). In particular, DNA double-strand break is a dangerous form of DNA damage with two main repair pathways: error-free homologous recombination and non-homologous end joining. In HR-deficient cells, cells attempt to repair DNA damage through non-homologous end joining, leading to increased total chromosomal rearrangement, overall genomic instability (2), and CNVs (25). Because of the intrinsic linkage between CNVs and HRD, a study was conducted to assess the importance of CNVs in predicting HRD.

In this study, we developed a novel biomarker from the view of the CNV landscape at subchromosomal and genetic resolutions, which provides a new perspective for studying HRD status in patients. We found that 8q24.2 amplification and 5q13.2 deletion were significantly associated with HRD status, whereas 19q12 amplification was markedly associated with non-HRD status in OV patients. This result was also validated in the AOCS cohort, suggesting that CNVs of these three chromosomal fragments were potential biomarkers for HRD in OV patients. Of note, we also found that the CNV of 8q24.2 was also applicable to other tumors for predicting HRD status. Further analysis of 8q24.2 showed that the amplification of two oncogenes, *MYC* and *NDRG1*, located on this fragment might be associated with HRD across tumor types.

In the pan-cancer analysis of HRD, we found that patients with ovarian serous cystadenocarcinoma, uterine carcinosarcoma, lung squamous cell carcinoma, sarcoma, esophageal carcinoma, stomach adenocarcinoma, bladder urothelial carcinoma, breast invasive carcinoma, and lung adenocarcinoma showed predominantly high mean HRD scores, which is consistent with the previously reported high frequency of HRD in ovarian and breast cancers (1) and confirms the universality of HRD. Regarding cross-tumor prediction studies of CNVs, 8q24.2 amplification was enriched, while 19q12 amplification most frequently occurred in OV and stomach adenocarcinoma, but 5q13.2 deletion was only reflected in OV. A possible explanation is that CNVs have different predictive sensitivity for HRD status in different tumors because of tumor heterogeneity and prior treatment. Compared to other biomarkers, CNVs may be able to extend the therapy targeting HRD to other tumors. Furthermore, we found that *MYC* amplification significantly correlated with HRD, which is consistent with the findings of Ning et al., who found that *MYC* or *MYCN* amplification yields sensitivity to PARP inhibitors through *Myc*-mediated transcriptional repression of *CDK18* (42). Consequently, we speculate that the other gene, *NDRG1*, may also influence homologous recombination through a mechanism that needs to be further investigated.

Owing to the lack of access to germline mutation data for the TCGA cohort, we used HRD score as an alternative grouping criterion, whereas HRR gene mutations were used as a grouping criterion in the AOCS cohort. We acknowledge that the results of these two criteria do not exactly coincide with patient grouping, but they are generalized to some extent. Each individual component of HRD score is markedly related to HRR gene mutations (17–19) and HRD score is also significantly associated with *BRCA* deficiency (43). Our results suggest that the CNVs of specific chromosomal fragments achieved satisfactory predictive performance in both cohorts, illustrating their consistency with the two major existing evaluation systems for HRD. Furthermore, the calculation of HRD score is based on high-density genome-wide single-nucleotide polymorphism array or next-generation sequencing genomic single-nucleotide polymorphism backbone probes, which have wide coverage but are not cost-effective, whereas the CNV biomarkers identified in our study for predicting HRD involve only three chromosomal fragments, which can be used as a complementary biomarker to evaluate HRD and provide patients with more options.

Our study has two limitations. First, this research is based on retrospective analysis, and further validation using both real-world data and even prospective studies are required before these CNV biomarkers can be truly applied in the clinical practice, such as CNVs predicting response to platinum-containing neoadjuvant or PARP inhibitor therapy. Second, the mechanism of the predictive role of CNVs for HRD was not investigated in depth in this study, and more basic experiments are needed to explore its underlying biological mechanism.

## Conclusion

In conclusion, this study provides evidence that CNVs in 5q13.2, 8q24.2, and 19q12 can predict HRD in OV patients. In addition, we confirmed a cross-tumor predictive role of 8q24.2 amplification for HRD. Further analysis of CNV in 8q24.2 at genetic resolution revealed that amplifications of the oncogenes located on this fragment, *MYC* and *NDRG1*, were also associated with HRD in a pan-cancer manner. The results of our analysis enrich HRD biomarkers at subchromosomal and genetic resolutions and stimulate the improvement of current clinical practice in detecting HRD by combining several levels and tests to comprehensively assess HRD status and provide more treatment options for patients.

## Data Availability Statement

The original contributions presented in the study are included in the article/**Supplementary Material**. Further inquiries can be directed to the corresponding authors.

## Author Contributions

MZ, ZY-D, and Q-XZ were responsible for the study design and final editing of the paper. MZ, S-CM, and J-LT drafted the manuscript. S-CM contributed to the bioinformatics and data analysis. JW and XB conducted the clinical evaluations. MZ, ZY-D, and Q-XZ checked the analysis and critically revised the manuscript. All authors contributed to the article and approved the submitted version.

## Funding

This study was supported by the National Natural Science Foundation of China (82171642, 81971332); and Sun Yat-Sen University Clinical Research 5010 Program (2016004).

## Conflict of Interest

The authors declare that the research was conducted in the absence of any commercial or financial relationships that could be construed as a potential conflict of interest.

## Publisher’s Note

All claims expressed in this article are solely those of the authors and do not necessarily represent those of their affiliated organizations, or those of the publisher, the editors and the reviewers. Any product that may be evaluated in this article, or claim that may be made by its manufacturer, is not guaranteed or endorsed by the publisher.

## Supplementary Material

Supplementary Figure 1 Flow chart of the study design.

Supplementary Figure 2 Histograms, quantile-quantile plots, and normality tests for the data.

Supplementary Table 1 Summary of dataset size.

Supplementary Table 2 Clinical characteristics of patients per HRD status from the TCGA-OV cohort.

Supplementary Table 3 HRD score of TCGA patients.

Supplementary Table 4 HRD status in AOCS cohort.

Supplementary Table 5 Summary of clinical characteristics of TCGA pan-cancer cohort.

Supplementary Table 6 Summary of cancer types and HRD score in TCGA pan-cancer cohort.

Supplementary Table 7 Pairwise comparisons of LOH/TAI/LST difference between groups stratified by the degree of amplification in 8q24.2 and 19q12.

## Abbreviations

HRD, homologous recombination deficiency; OV, ovarian cancer; PARP, poly ADP ribose polymerase; HRR, homologous recombination repair; TAI, telomeric-allelic imbalance; LOH, loss of heterozygosity; LST, large-scale state transitions; CNV, copy number variation; aCGH, array comparative genomic hybridization; AOCS, Australian Ovarian Cancer Study; TCGA, The Cancer Genome Atlas; ICGC, International Cancer Genome Consortium; GDC, Genomic Data Commons; GISTIC, genomic identification of significant targets in cancer; ROC, receiver operating characteristic; AUC, area under the ROC curve; FDR, false discovery rate.
