# Supplementary figures and images for: Inferring Homologous Recombination Deficiency of Ovarian Cancer From the Landscape of Copy Number Variation at Subchromosomal and Genetic Resolutions
Source: Front Oncol. 2021 Dec 16;11:772604. doi: 10.3389/fonc.2021.772604 (PMC8716765; doi:10.3389/fonc.2021.772604)

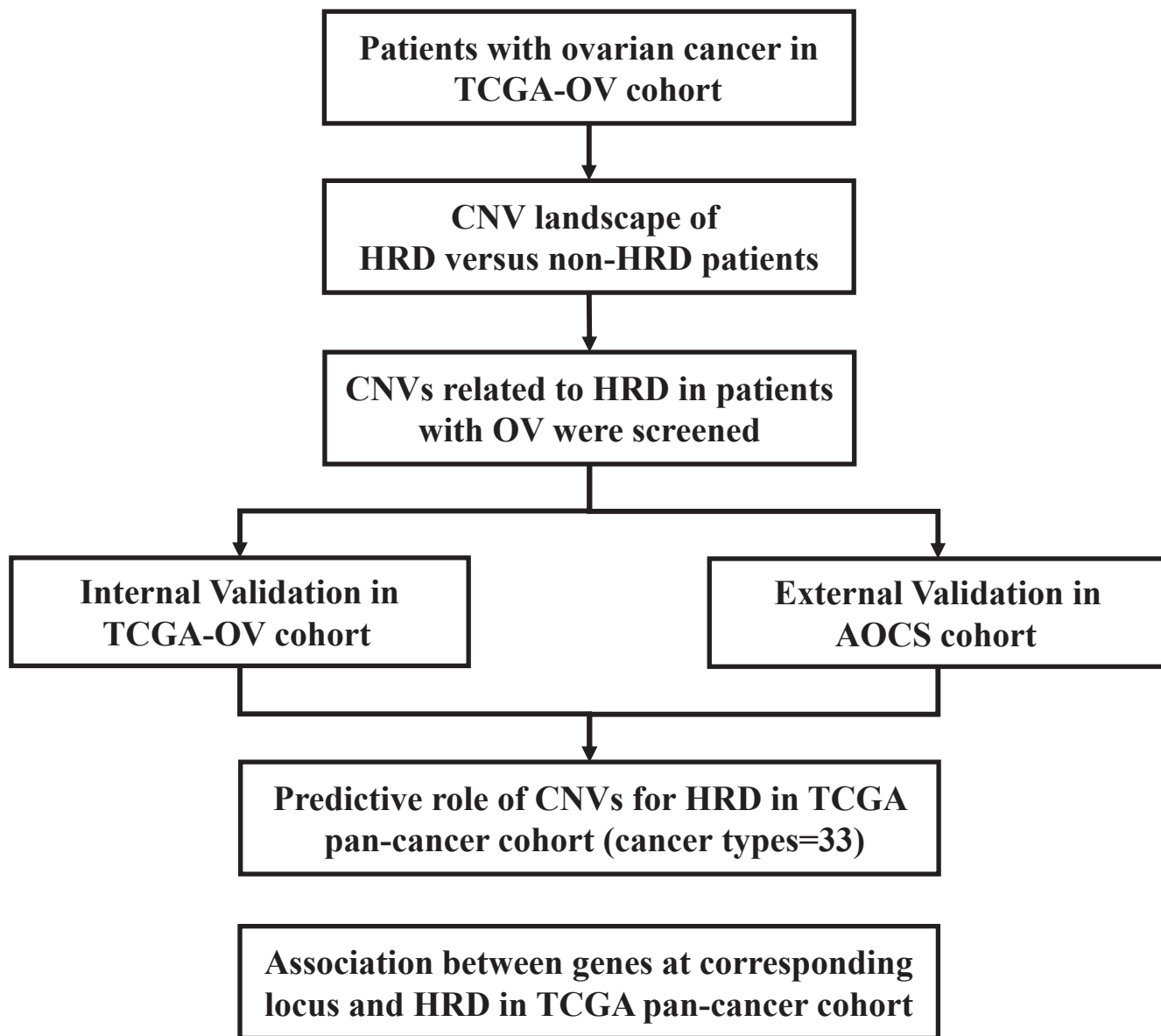

Supplement: Supplementary Figure 1 — Flow chart of the study design. [file DataSheet_1.pdf]
