# Supplementary material for: Inferring Homologous Recombination Deficiency of Ovarian Cancer From the Landscape of Copy Number Variation at Subchromosomal and Genetic Resolutions
Source: Front Oncol. 2021 Dec 16;11:772604. doi: 10.3389/fonc.2021.772604 (PMC8716765; doi:10.3389/fonc.2021.772604)

p value: Kolmogorov-Smirnov normality test
(the null hypothesis: the data distribution is normal)

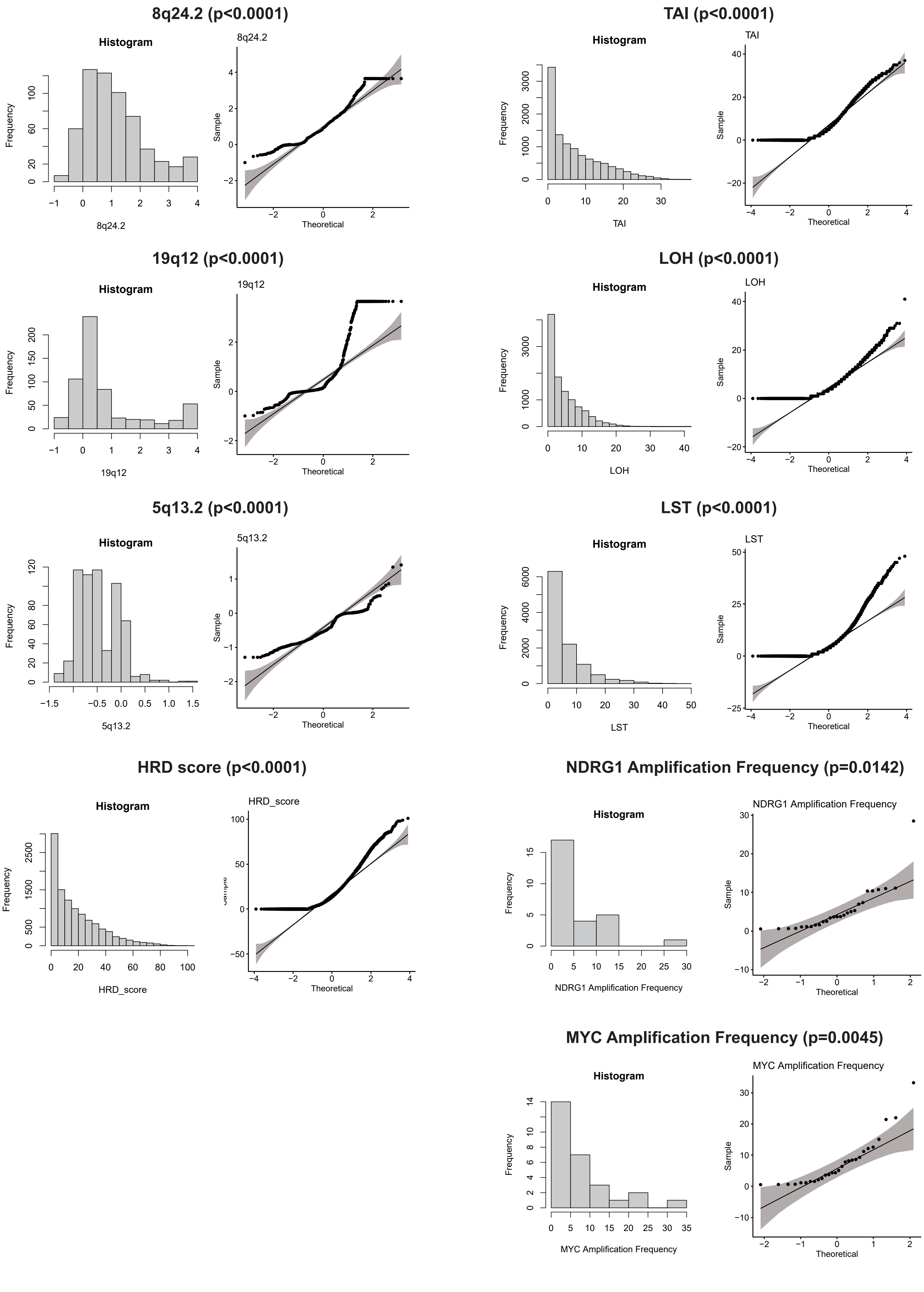

Supplement: Supplementary Figure 2 — Histograms, quantile-quantile plots, and normality tests for the data. [file DataSheet_2.pdf]
