# Supplementary material for: Inferring Homologous Recombination Deficiency of Ovarian Cancer From the Landscape of Copy Number Variation at Subchromosomal and Genetic Resolutions
Source: Front Oncol. 2021 Dec 16;11:772604. doi: 10.3389/fonc.2021.772604 (PMC8716765; doi:10.3389/fonc.2021.772604)
Supplement: Supplementary Table 1 — Summary of dataset size. [file Table_1.docx]

**Table S1.** Summary of dataset size.

| Datasets | # Samples | # Patients |
| --- | --- | --- |
| TCGA-OV |  |  |
| CNV data | 597 | 582 |
| Clinical data | NA | 587 |
| HRD status | 559 | 559 |
| CNV+Clinical+HRD | NA | 559 |
| TCGA-PANCAN |  |  |
| CNV data | 10,741 | 10,712 |
| Clinical data | NA | 11,167 |
| HRD status | 10,647 | 10,585 |
| CNV+Clinical+HRD | NA | 10,560 |
| AOCS |  |  |
| CNV data | 93 | 93 |
| Clinical data | NA | 93 |
| HRD status | 80 | 80 |
| CNV+Clinical+HRD | NA | 80 |

Abbreviation: CNV, copy number variation; HRD, Homologous recombination deficiency; NA, not applicable.
