# Supplementary material for: Inferring Homologous Recombination Deficiency of Ovarian Cancer From the Landscape of Copy Number Variation at Subchromosomal and Genetic Resolutions
Source: Front Oncol. 2021 Dec 16;11:772604. doi: 10.3389/fonc.2021.772604 (PMC8716765; doi:10.3389/fonc.2021.772604)
Supplement: Supplementary Table 2 — Clinical characteristics of patients per HRD status from the TCGA-OV cohort. [file Table_2.docx]

**Table S2.** Clinical characteristics of patients per HRD status from the TCGA-OV cohort.

| Clinical characteristics | Non-HRD | HRD | P-value |
| --- | --- | --- | --- |
| Primary diagnosis |  |  | 0.2653 |
| Cystadenocarcinoma, NOS | 1 | 0 |  |
| Papillary serous cystadenocarcinoma | 3 | 1 |  |
| Serous cystadenocarcinoma, NOS | 242 | 310 |  |
| Serous surface papillary carcinoma | 0 | 1 |  |
| Stage |  |  | 0.0570 |
| IA | 0 | 2 |  |
| IB | 0 | 3 |  |
| IC | 2 | 9 |  |
| IIA | 3 | 1 |  |
| IIB | 2 | 3 |  |
| IIC | 5 | 16 |  |
| IIIA | 2 | 5 |  |
| IIIB | 9 | 16 |  |
| IIIC | 188 | 206 |  |
| IV | 32 | 50 |  |
| Not reported | 3 | 1 |  |
| Ethnicity |  |  | 0.6143 |
| Hispanic or Latino | 3 | 7 |  |
| Not Hispanic or Latino | 146 | 176 |  |
| Not reported | 97 | 129 |  |
| Race |  |  | 0.3156 |
| American Indian or Alaska native | 1 | 2 |  |
| Asian | 5 | 14 |  |
| Black or African American | 14 | 20 |  |
| Native Hawaiian or other pacific islander | 0 | 1 |  |
| White | 217 | 256 |  |
| Not reported | 9 | 19 |  |
| Treatment or therapy |  |  | 0.2047 |
| No | 111 | 164 |  |
| Yes | 127 | 140 |  |
| Not reported | 8 | 8 |  |
