# Supplementary material for: Inferring Homologous Recombination Deficiency of Ovarian Cancer From the Landscape of Copy Number Variation at Subchromosomal and Genetic Resolutions
Source: Front Oncol. 2021 Dec 16;11:772604. doi: 10.3389/fonc.2021.772604 (PMC8716765; doi:10.3389/fonc.2021.772604)
Supplement: Supplementary Table 7 — Pairwise comparisons of LOH/TAI/LST difference between groups stratified by the degree of amplification in 8q24.2 and 19q12. [file Table_7.docx]

**Table S7.** Pairwise comparisons of LOH/TAI/LST difference between groups stratified by the degree of amplification in 8q24.2 and 19q12.

|  | P (High Amp vs Low Amp) | P (Low Amp vs Normal copy) | P (High Amp vs Normal copy) |
| --- | --- | --- | --- |
| 8q24.2 |  |  |  |
| LOH | 0.0110 | 0.0006 | <0.0001 |
| TAI | 0.0004 | 0.0003 | <0.0001 |
| LST | <0.0001 | <0.0001 | <0.0001 |
| 19q12 |  |  |  |
| LOH | <0.0001 | 0.18 | <0.0001 |
| TAI | <0.0001 | 0.037 | 0.0031 |
| LST | <0.0001 | 0.35 | <0.0001 |

P values were calculated by the Wilcoxon signed rank test. Abbreviation: LOH, loss of heterozygosity; TAI, telomere-allelic imbalance; LST, large-scale state transition; Amp: amplification.
